# Tumor-Infiltrating Natural Killer Cell Characterization in Pancreatic Ductal Adenocarcinoma

**DOI:** 10.3390/cells15090797

**Published:** 2026-04-28

**Authors:** Andreia Maia, Hasti Calá, Eric de Sousa, Joana R. Lérias, Carolina M. Gorgulho, Patrícia A. António, Jéssica Kamiki, Dário Ligeiro, Luis M. Borrego, Markus Maeurer, Mireia Castillo-Martin

**Affiliations:** 1 Molecular and Experimental Pathology Laboratory, Champalimaud Centre for the Unknown, Champalimaud Foundation, 1400-038 Lisbon, Portugal; andreiaf_maia@dfci.harvard.edu (A.M.); hasti.cala@research.fchampalimaud.org (H.C.); 2 ImmunoSurgery/ImmunoTherapy Laboratory, Champalimaud Centre for the Unknown, Champalimaud Foundation, 1400-038 Lisbon, Portugal; eric.desousa@research.fchampalimaud.org (E.d.S.); joana.lerias@research.fchampalimaud.org (J.R.L.); carolina.gorgulho@research.fchampalimaud.org (C.M.G.); patricia.antonio@research.fchampalimaud.org (P.A.A.); jessica.kamiki@research.fchampalimaud.org (J.K.); dario.ligeiro@fundacaochampalimaud.pt (D.L.); markus.maeurer@fundacaochampalimaud.pt (M.M.); 3 NOVA Medical School, NOVA University of Lisbon, 1099-085 Lisbon, Portugal; 4 Comprehensive Health Research Centre (CHCR), NOVA Medical School, NOVA University of Lisbon, 1099-085 Lisbon, Portugal; luis.borrego@nms.unl.pt; 5 Immunoallergy Department, Hospital da Luz, 1600-209 Lisbon, Portugal; 6 I Medical Clinic, University of Mainz, 55122 Mainz, Germany; 7 Pathology Service, Champalimaud Clinical Centre, Champalimaud Foundation, 1400-038 Lisbon, Portugal

**Keywords:** solid tumours, pancreatic ductal adenocarcinoma, adoptive cell therapy, tumor-infiltrating lymphocytes, TIL therapy, natural killer cells, NK-TIL

## Abstract

**Highlights:**

This pilot study is the first to comprehensively characterize NK cell subsets from both peripheral blood and matched tumor specimens of PDAC patients. Our preliminary results demonstrate that despite the immunosuppressive nature of the PDAC microenvironment, NK cells not only persist but can be effectively expanded and activated ex vivo.

**What are the main findings?**

- Distinct distribution of NK cells within the PDAC tumor microenvironment, with higher infiltration in peripheral regions.
- Superior expansion rates and tumor infiltration markers in TIL-derived NK cells compared to peripheral blood NK cells.
- Enhanced activation and cytotoxic profiles of patient-derived NK cells following IL-2, IL-15, and brief IL-12 stimulation.

**Abstract:**

Pancreatic ductal adenocarcinoma (PDAC) has high mortality rates, poor prognosis, and currently limited effective treatments. Natural killer (NK) cells from tumor-infiltrating lymphocytes (TIL) show promise for cancer treatment due to their ability to migrate to the tumor microenvironment (TME) and safe profile. However, expanding functional patient-derived NK cells remains challenging. Here, we cultured, expanded, and characterized TIL-NK cells isolated from central and peripheral tumor regions from PDAC. Ex vivo patient-derived PBMCs and TIL were cultured under IL-2, IL-15, and IL-12 stimulation. Phenotypical and functional NK cell characterization was assessed at the time of surgery and after 12 days of culture evaluating immunophenotype, expansion rate, and activation. A distinct distribution of NK cell infiltration was observed within the TME, with higher NK cell numbers in the periphery of the tumor compared to the central area. Most NK cells displayed a cytotoxic phenotype (CD56^+^ CD16^+^). Compared to PBMCs, TIL-NK cells expressed lower activation markers but superior tumor infiltration and expansion rates, particularly those isolated from the central regions. Notably, cytokine stimulation improved patient-derived NK cell activation and cytotoxic profile. This pilot study provides preliminary but critical insights regarding TIL-NK cells from PDAC patients, laying groundwork for developing NK cell-based immunotherapies for solid tumors.

## 1. Introduction

Pancreatic ductal adenocarcinoma (PDAC) is among the most aggressive solid malignancies worldwide, characterized by high mortality rates due to its late diagnosis, chemoresistance, and immunosuppressive tumor microenvironment (TME) [1,2]. Surgical resection combined with chemotherapy (CT) remains the standard-of-care treatment [1,2,3], although most patients relapse and frequently fail to respond to different CT regimens, highlighting the need for more effective therapeutic strategies [4,5].

Adoptive cell transfer therapy, including tumor-infiltrating lymphocytes (TIL) therapy, has demonstrated specific tumor lysis and cytokine secretion, particularly in hematological malignancies [6,7,8]. TIL therapy has shown modest efficacy in solid tumors although with promising results in metastatic breast and cervical cancer [9,10]. Ongoing clinical trials (NCT05098197 and NCT03935893) are investigating the safety and efficacy of TIL therapy in PDAC. However, challenges such as downregulation of major histocompatibility complex class I (MHC-I) molecules in PDAC hinder T-cell recognition and impact treatment efficacy, emphasizing the need for therapies that operate independently of MHC-I [11,12,13,14].

Natural killer (NK) cells offer a promising alternative as they can recognize malignant cells independently of MHC-I molecules and do not require prior sensitization to specific antigens [15]. NK cells are categorized based on CD56 and CD16 expression into regulatory cells (CD56^bright^ CD16^−^), intermediate cells (CD56^+^ and CD16^−^), and cytotoxic cells (CD56^+^ CD16^+^) [16,17,18,19,20]. Due to their cytotoxic properties, NK cell-based therapy has shown promising results in hematological malignancies and is being evaluated for solid tumors [21,22,23]. NK cell-based immunotherapy is notable for its lack of severe cytokine release syndrome, neurotoxicity, and the absence of graft-versus-host disease, which are common side effects of T-cell TIL therapy [24,25,26].

Despite NK cell potential, achieving clinically relevant numbers remains challenging [23,27]. While various expansion methods have been reported, there is no consensus on the optimal protocol, particularly for TIL-derived NK cells [28]. The lack of information on TIL-NK cell expansion from PDAC is partly due to the difficulty in obtaining fresh tumor specimens with minimal ischemia time from treatment-naïve patients and also due to it being described as a “cold” tumor [29].

To address these challenges, we developed a protocol for isolating and expanding TIL-NK cells from fresh PDAC specimens and corresponding peripheral blood (PB). Despite a limited patient cohort, this study reveals preliminary and essential insights into the distinct distribution and characterization of NK cells within the PDAC TME and represents a crucial step towards advancing NK cell-based immunotherapy for PDAC and potentially other solid malignancies.

## 2. Materials and Methods

### 2.1. Patient Selection

**Figure 1:**
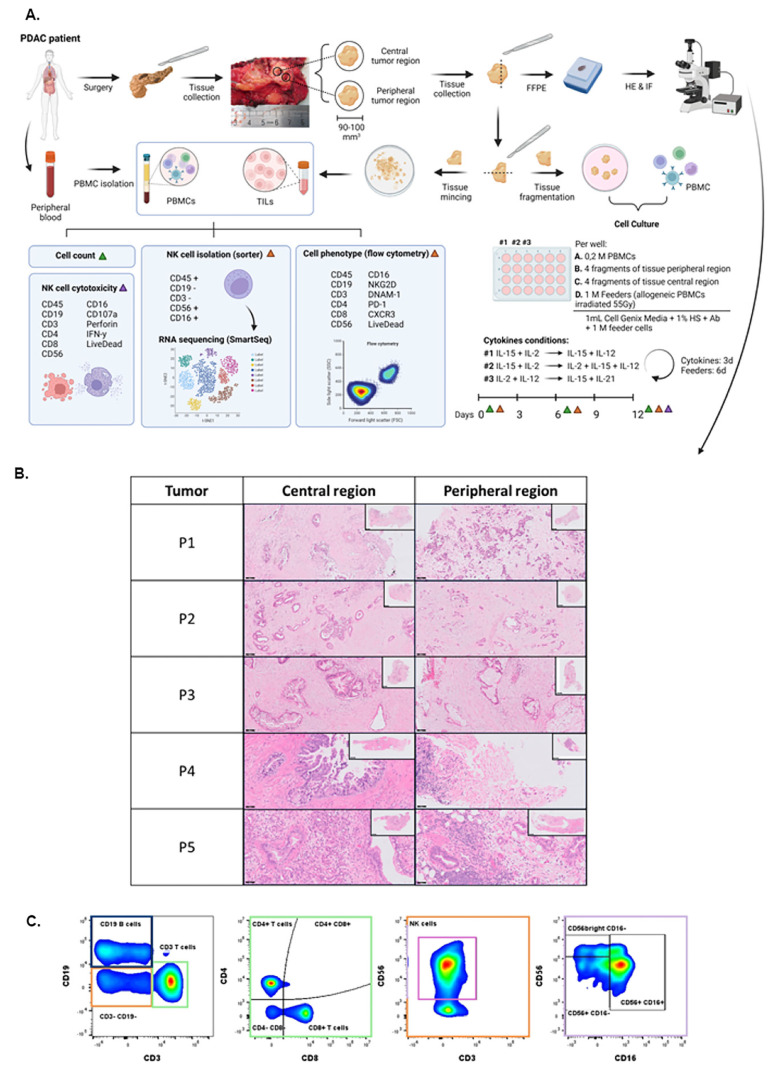
Methodology overview for NK cell isolation and characterization from fresh PDAC tumor specimens and peripheral blood. (**A**) Schematic representation of the methodology for isolating and characterizing natural killer (NK) cells from peripheral blood mononuclear cells (PBMCs) and tumor-infiltrating lymphocytes (TIL). Fresh tumor specimens were obtained from central (TIL-C) and peripheral (TIL-P) tumor regions, along with peripheral blood (PB) from patients with PDAC. Tumor specimens were divided into three fragments: one for formalin-fixed paraffin-embedded (FFPE) block preparation, subsequent hematoxylin and eosin (HE) and multiplex immunofluorescence (IF) staining,. The remaining two fragments were used for cell culture and NK cell phenotypic and functional characterization alongside PBMCs isolated at the time of surgery. NK cells were cultured with 55Gγ-irradiated allogeneic PBMC feeder cells in media supplemented with IL-2 and IL-15 for 6 days, followed by media supplemented with IL-2, IL-12, and IL-15, for an additional 6 days. Fresh media was added every 3 days, and feeder cells were replenished on day 6. Cell counts and NK cell immunophenotype were assessed on days 0, 6, and 12 by flow cytometry, measuring CD45, CD19, CD3, CD4, CD8, CD56, CD16, NKG2D, DNAM-1, PD-1, and CXCR3 frequency. RNA sequencing analysis was performed on flow-sorted NK cells at days 0 and 12. NK cell cytotoxicity was evaluated at the end of the cell culture by measuring IFN-γ, perforin, and CD107a expression using flow cytometry. (**B**) Histology images of central and peripheral tumor regions from the collected PDAC specimens. Scale bars correspond to 50 µm and 2 mm (inserts). (**C**) Flow cytometry gating strategy used for phenotypic characterization of NK cells and other lymphocytes.

Five patients who underwent surgery at the Champalimaud Clinical Centre for PDAC with no neoadjuvant CT and whose tumors were grossly identifiable were selected for this study. Blood from these patients was collected right before surgery and peripheral blood mononuclear cells (PBMCs) were isolated and processed as described below and illustrated in Figure 1A. Fresh tissue was obtained from the Anatomic Pathology Service with an ischemia time of <20 min through the Champalimaud Foundation Biobank (CFB). The tumor area was grossly identified by a specialized pathologist by palpation, sectioned through the largest diameter, and then two 90 to 100 mm^3^ fragments were collected using a 4 mm dermatological punch: one from the middle of the palpable nodule—identified as the central tumor region (CTR) and another from the edge of the nodule, near the surrounding normal parenchyma—identified as the peripheral tumor region (PTR). Samples were processed immediately after the procedure as illustrated in Figure 1A. All patients had signed the CFB informed consent and the study was approved by Institutional Ethics Committees.

### 2.2. PBMCs Isolation and Culture

PBMCs were isolated from one 9 mL EDTA tube using the Ficoll density gradient centrifugation technique. Subsequently, 2 × 10^5^ PBMCs were cultured with 1 × 10^6^ previously 55Gγ-irradiated allogeneic PBMCs feeder cells in 24-well plates for 12 days, incubated at 37 °C with 5% CO^2^. Each well contained 1 mL of media (Cell Genix GMP #20801-0500, and multiplex immunofluorescence (mIF) Götingen, Germany) with 10% human serum (Sigma-Aldrich #H4522, Burlington, MA, USA), 1% Penicillin/Streptomycin (Corning #30-002, Tewksbury, MA, USA), 1 µg/mL Clindamycin (Fresenius Kabi #10030860, Bad Homburg vor der Höhe, Germany), 1 µg/mL Amphotericin (Sigma-Aldrich #A2942, Burlington, MA, USA) and 10 µg/mL Ciprofloxacin (Fresenius Kabi #10071960, Bad Homburg vor der Höhe, Germany), as well as IL-2 (1000 IU/mL, Miltenyi #130-097-746, Bergisch Gladbach, Germany), IL-12 (10 IU/mL, Miltenyi #130-096-798, Bergisch Gladbach, Germany), and IL-15 (180 IU/mL, Miltenyi #130-095-765, Bergisch Gladbach, Germany). Every 3 days, fresh medium with cytokines was added, while feeder cells were added every 6 days. In previous experiments from our group, over 29 cytokine combinations comprising IL-2, IL-12, IL-15 and IL-18 were evaluated in healthy donors’ and patients’ PBMCs to assess the optimal cocktail for NK cells expansion and based on these analyses, for this study NK cells were incubated with IL-2 and IL-15 for 6 days, followed by IL-2, IL-15, and IL-12 for another 6 days.

### 2.3. Tumor Processing and TIL Isolation

Each specimen from CTR and PTR was divided into three fragments (Figure 1A).

One fragment was fixed with 10% buffered formalin and embedded in paraffin to generate a formalin-fixed paraffin-embedded (FFPE) block for posterior hematoxylin and eosin (H&E) and multiplex immunofluorescence (mIF) staining. The remaining two fragments were subdivided into 1–2 mm^3^ fragments. One piece was minced and used for flow cytometry to assess TME immune cell composition at the time of the surgery (day 0), while the others were cultured in 24-well plates (2–3 pieces/well) under the same conditions described for PBMCs. After 6 days, tumor pieces were carefully removed.

### 2.4. Immunophenotyping

The immunophenotype of TIL and PBMCs was assessed on days 0, 6, and 12 of culture using flow cytometry (Cytoflex LX cytometer, Beckman Coulter, Indianapolis, IN, USA). The surface markers used are summarized in Table 1. Antibodies were incubated in the dark for 15 min at 4 °C. The gating strategy to identify the PBMCs and TIL phenotype is represented in Figure 1C.

### 2.5. Functional Assays

Cell cytotoxicity was assessed by measuring CD107a protein externalization, and IFN-γ and perforin production. PBMCs and TIL were cultured with K562 cells (ratio 10:1) for 6 h at 37 °C. CD107a antibody (see Table 1) was added during incubation. After 1 h, GolgiStop/Monensin (BD #554724, Franklin Lakes, NJ, USA) and 20 µg/mL Brefeldin A (BioLegend #420601, San Diego, CA, USA) were added. As positive control, PBMCs were stimulated with 1 µg/mL Phorbol myristate acetate (Merck #P1585-1 mg, Rahway, NJ, USA) and 40 µg/mL ionomycin (ThermoFisher #I24222, Waltham, MA, USA). Unstimulated PBMCs were used as negative controls. Subsequently, cells were stained with the immunophenotyping antibodies highlighted in grey in Table 1 for 15 min at 4 °C. Then, cells were permeabilized with Perfix-nc fixative reagent (Beckman Coulter, #B31167, Indianapolis, IN, USA) for 15 min at room temperature (RT), followed by anti-IFN-γ and anti-perforin (Table 1) staining for 45 min in the dark.

### 2.6. Multiplex IF Staining (mIF)

mIF was performed using 5 µm thick FFPE slides. After deparaffinization, slides were submitted to Epitope Retrieval Buffer pH9 (Leica Biosystems #RE7119, Deer Park, IL, USA) for 30 min and afterwards incubated with blocking solution (2% BSA-1X PBS) for 20 min at RT. Then, slides were incubated with the first set of primary antibodies (Rat anti-CD3 IgG [CD3-12], Mouse anti-CD16 IgG2a [2H7], and Rabbit anti-NKG2D IgG [EPR24072-342]) followed by the corresponding secondary antibodies (Donkey anti-rat IgG Alexa Fluor^TM^ 594 (ThermoFisher #A21209, Waltham, MA, USA), Donkey anti-Mouse IgG Alexa Fluor^TM^ 568 (ThermoFisher #A10037, Waltham, MA, USA), and Zenon rabbit IgG labeling kit Alexa Fluor 647 (ThermoFisher #Z25308, Waltham, MA, USA), Goat anti-cytokeratin 18 (CK18) IgG antibody followed by its secondary (Donkey anti-goat IgG Alexa Fluor 555 (ThermoFisher # A21432, Waltham, MA, USA) and finally with Alexa Fluor^TM^ 488 rabbit anti-NCAM1 (CD56) IgG [EP2567Y] (Table 1). All primary antibodies were incubated for 2 h and secondaries for 1 h at RT. Slides were counterstained with ProLong Gold antifade reagent with DAPI (Invitrogen #P36935, Waltham, MA, USA) and visualized under a Nikon90i (Nikon, Shinagawa-ku, Tokyo, Japan) fluorescence microscope equipped with a multispectral camera (CRI).

### 2.7. RNA Sequencing

Full-length cDNAs were generated from total RNA, following the published SMART-Seq2 protocol [30]. Sequencing was carried out on the NextSeq 2000 system (Illumina, San Diego, CA, USA) with 100 bp single-end reads. STAR was used to map the reads to the human genome version GRCh [31] obtained from Ensembl release 11069 and to count the number of reads per gene [32]. DESeq2 was used to estimate differential expression [33]. g:Profiler with default parameters except with the exclusion of GO annotations was used for functional enrichment analysis [34].

### 2.8. Data Analysis and Statistics

Flow cytometry data was analyzed with FlowJo v10 and Excel. mIF images were generated with the Nuance software (version 3.0.2) and analyzed using Fiji/Image J software. The number of NK cells per mm^2^ was quantified by dividing the number of cells by the tissue area. Differential expression from sequencing data was estimated in R version 4.1.2 with DESeq2, considering only genes with a log2Fold change. Statistical analyses were performed using GraphPad Prism 10.1.1 software. The normality of the data was assessed using the Shapiro–Wilk test due to the small sample size. One-way ANOVA or the Friedman test was used to compare differences between PBMC-, TIL-C-, and TIL-P-NK cells at the time of the surgery, followed by Tukey’s multiple comparison test. Two-way ANOVA was used to compare differences between PBMC-, TIL-C-, and TIL-P-derived NK cells during cell culture, followed by Tukey’s multiple comparison test. A paired *t*-test or Wilcoxon test was used to compare NK cell numbers/mm^2^ between TIL-C and TIL-P samples. Data were summarized in graphs and were expressed as mean ± SD. *p*-value symbols correspond to * *p* < 0.05, ** *p* < 0.01, *** *p* < 0.001 and **** *p* < 0.0001.

## 3. Results

### 3.1. Patient Characteristics

Clinico-pathological characteristics are summarized in Table 2. They were two males and three females aged between 67 and 90 at the time of surgery. All patients were diagnosed with stage II or III PDAC, and four associated with intraductal papillary mucinous neoplasm (IPMN). All tumors showed mismatch repair proteins expression by immunohistochemistry, did not express HER2, and did not harbour BRAF or KRAS mutations. Patients P2, P4, and P5 are alive without disease (AWOD), patient P3 is alive with disease (AWD), presenting metastasis in the liver and peritoneum 16 months after surgery, and patient P1 died of disease (DOD) 15 months after surgery.

### 3.2. Distinct Tumor Region TIL-NK Infiltration

The methodology for isolating and characterizing PBMCs and TIL is illustrated in Figure 1A. The presence of PDAC in the collected specimens was confirmed by histological analyses (Figure 1B).

**Figure 2:**
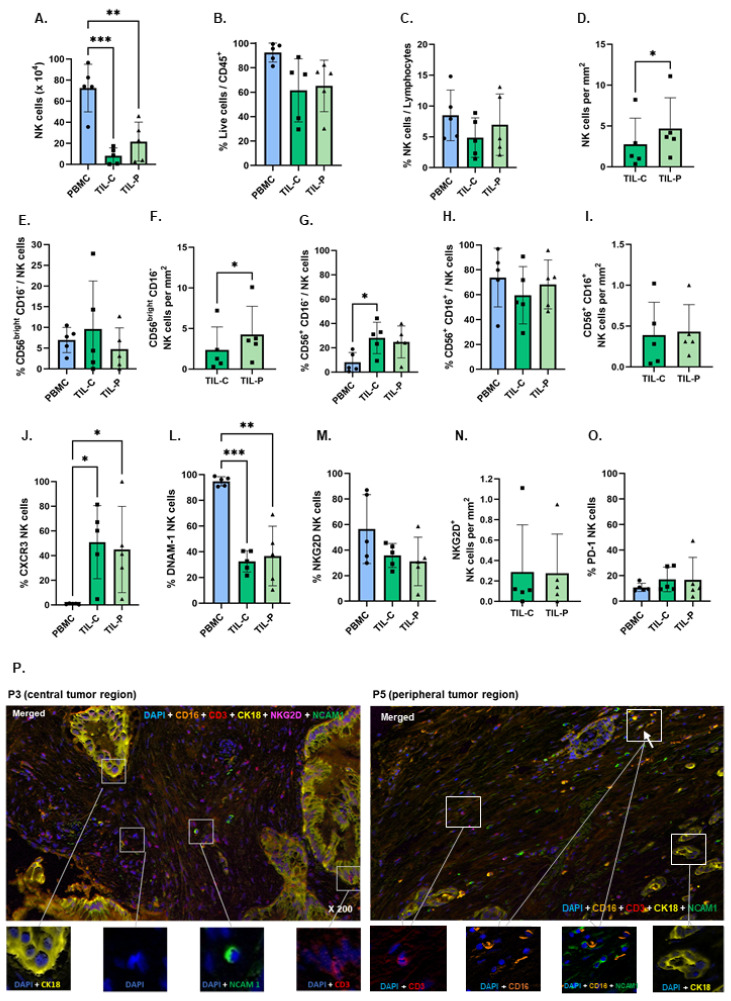
Characterization of PBMC- and TIL-NK cells from central (TIL-C) and peripheral (TIL-P) PDAC tumor regions at surgery. (**A**) Absolute NK cell numbers in 9 mL of peripheral blood and fresh tumor specimens. (**B**) Viability of CD45^+^ cells. (**C**) Percentage and (**D**) cell numbers per mm^2^ of NK cells. (**E**) Percentage and (**F**) cell number per mm^2^ of regulatory (CD56^bright^ CD16^−^) NK cells. (**G**) Percentage of intermediate (CD56^+^ CD16^−^) NK cell subset among total NK cells. (**H**) Percentage and (**I**) cell number per mm^2^ of cytotoxic (CD56^+^ CD16^+^) NK cells. Percentage of (**J**) CXCR3, (**L**) DNAM-1, and (**M**) NKG2D expression in NK cells. (**N**) NKG2D^+^ NK cell numbers per mm^2^ of tumor specimens. (**O**) Percentage of PD-1 expression in NK cells. (**P**) Representative multiplex immunofluorescence (mIF) images of PDAC tissue specimens (×200), stained with CD16 (Alexa Fluor 568), CD3 (Alexa 594), NKG2D (Alexa Fluor 647), cytokeratin 18 (CK18, Alexa Fluor 555), NCAM-1/CD56 (Alexa Fluor 488), and DAPI (blue). Arrow in right panel points to two CD56^+^ CD16^+^ NK cells. Graphs represent the mean and SD (*n* = 5); PBMCs are represented by blue bars, TIL-C by darker green, and TIL-P by lighter green bars. Statistical differences were assessed using one-way ANOVA (**A**,**B**,**C**,**E**,**G**,**H**,**J**,**L**,**M**), paired *t*-test (**D**,**F**,**I**,**N**), or Friedman test (**O**), followed by Tukey’s multiple comparison test; * *p* ≤ 0.05, ** *p* ≤ 0.01, and *** *p* ≤ 0.001.

PBMCs contained an average of 72.5 × 10^4^ NK cells per 9 mL blood tube (Figure 2A), whereas an average of 8.0 × 10^4^ and 21.5 × 10^4^ NK cells was identified in TIL-C and TIL-P, respectively (Figure 2A and Figure S1A). The viability of patient-derived PBMCs was superior to TIL, although approximately 60% of TIL remained viable (Figure 2B). As expected, NK cells represent a small fraction of the CD45^+^ cells, with PBMCs displaying an average of 8.5% NK cells, and TIL-C and TIL-P presenting 4.9% and 7.0%, respectively (Figure 2C), suggesting higher NK cell infiltration in the PTR. Immunofluorescence (mIF) in tumor tissue further confirmed higher numbers of NK cells in PTR than CTR (Figure 2D). Notably, PDAC with a higher stage (patients P3 and P5) exhibited a specific pattern, with a higher percentage of NK cells infiltrating the CTR (Figure S1B,C), suggesting a potential shift in NK cell distribution with disease progression.

An in-depth characterization of NK cell subsets revealed a slightly higher fraction of regulatory NK cells (CD56^bright^ CD16^−^) in TIL-C (9.6%) compared to TIL-P (4.8%) and PBMCs (7.0%) (Figure 2E). However, the absolute number of regulatory NK cells was lower in TIL-C (2.4 cells/mm^2^) compared to TIL-P (4.3 cells/mm^2^) (Figure 2F), which correlates with superior total NK cells in the PTR (Figure 2A). Additionally, clinically more aggressive PDAC (patients P1 and P3) displayed a higher percentage of regulatory NK cells in the CTR compared to the PTR, with patient P3 also showing increased total cell numbers (Figure S2A,B). The intermediate NK cell subset (CD56^+^ CD16^−^) was significantly more prevalent in the TIL-C (28.1%) and TIL-P (24.8%) compared to PBMCs (8.0%) (Figure 2G and Figure S2C), suggesting potential CD16 downregulation in the TME. Cytotoxic NK cells (CD56^+^ CD16^+^) remained the most frequent NK cell subset across all samples but were slightly less prevalent in TIL-C (59.6%) (Figure 2H,I and Figure S2D,E).

These findings indicate superior NK cell infiltration in the PTR. Interestingly, more advanced PDAC showed NK cell enrichment in the tumor center. Regarding NK subsets, cytotoxic NK cells are predominant in general, but CTR exhibit increased CD16^−^ NK cells, particularly in more aggressive tumors. This suggests that NK cells infiltrating CTR may have superior tumor infiltration ability but potentially reduced cytotoxicity.

### 3.3. Functional Impairment of TIL-NK Cells

NK cell function was assessed by the immunophenotyping of cell surface markers related to cell trafficking (CXCR3), cell activation (DNAM-1 and NKG2D), and exhaustion (PD-1).

TIL-C and TIL-P-N cells showed significantly higher levels of CXCR3 (50.9% and 45.0%, respectively) compared to PBMC-NK cells (0.8%) (Figure 2J). In patients with more advanced stages (P3 and P5), TIL-C showed enhanced CXCR3 expression compared to TIL-P (Figure S3A), which correlates with a higher number of NK cells (Figure S1B), indicating superior infiltration capacity. PBMC-NK cells exhibited significantly higher DNAM-1 expression (94.8%) compared to TIL-C (32.5%) and TIL-P (36.7%) (Figure 2L). PBMC-NK cells also displayed superior NKG2D expression (56.5%) compared to TIL-NK cells (~30%) (Figure 2M and Figure S3C), but similar numbers of NKG2D^+^ NK cells/mm^2^ were observed in TIL-NK cells (Figure 2N,P). The exhaustion marker PD-1 was expressed at low levels across all NK cell sources, with PBMCs showing the lowest percentage (10.8% versus 17.1% and 16.8% in TIL-C and TIL-P, respectively; Figure 2O).

Overall, these observations suggest that TIL-NK cells exhibit superior tumor infiltration capacity but are potentially less activated than PBMC-NK cells, with no significant differences between CTR and PTR.

### 3.4. TIL-NK Cells from CTR Show Superior Expansion and Proliferation

**Figure 3:**
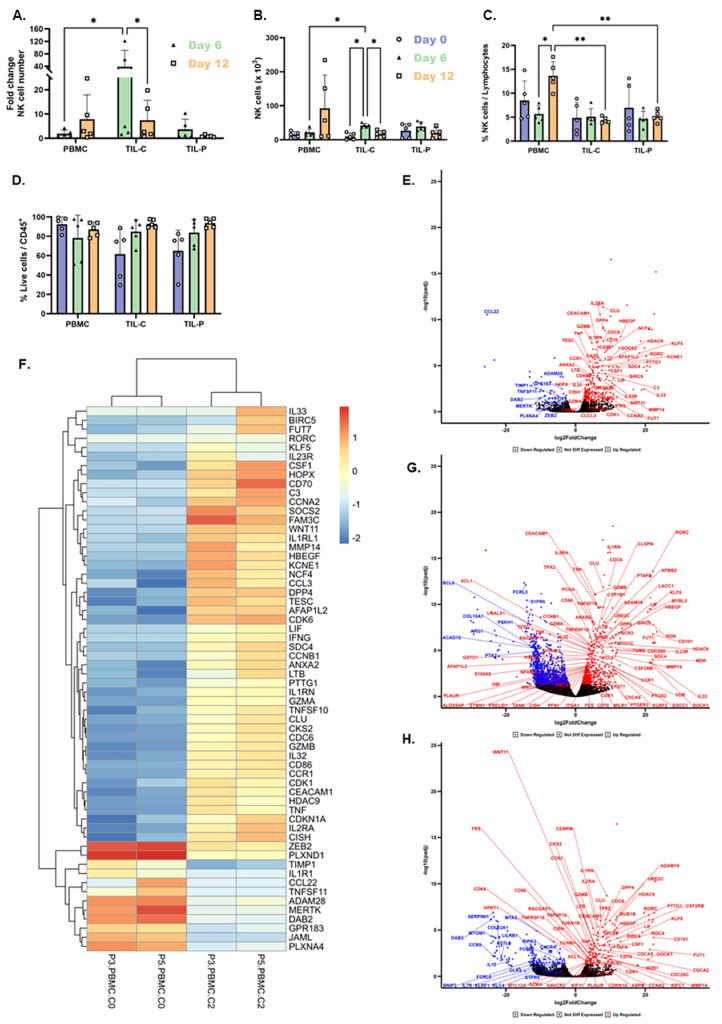
Expansion rate, proliferation capacity, and transcriptional changes in patient-derived NK cells from PBMCs and TIL. (**A**) NK cell fold change during ex vivo expansion, (**B**) absolute NK cell numbers, and (**C**) percentage of NK cells in total lymphocytes. (**D**) Viability of CD45^+^ cells throughout the culture period. (**E**) Volcano plot and (**F**) heatmap illustrating differentially expressed genes in PBMC-derived NK cells cultured under IL-2, IL-15, and brief IL-12 stimulation compared to NK cells at the time of the surgery (PBMC-NK cells). (**G**) Volcano plot showing differentially expressed genes in NK cells from TIL-C cultured under IL-2, IL-15, and brief IL-12 stimulation, compared to NK cells at the time of the surgery (PBMC-NK cells). (**H**) Volcano plot depicting differentially expressed genes in NK cells from TIL-P cultured under IL-2, IL-15, and brief IL-12 stimulation, compared to NK cells at the time of the surgery (PBMC-NK cells). Each bar in the graphs represents the mean and SD from 5 samples measured at day 0 (purple), day 6 (green), and day 12 (orange). Statistical differences were assessed using ANOVA (* *p* ≤ 0.05 and ** *p* ≤ 0.01). RNA sequencing was performed on flow-sorted NK cells from patient-derived PBMC and TIL (*n* = 2).

TIL-C displayed the highest expansion rate on day 6, with an average of 38.4-fold, reaching 42.0 × 10^3^ NK cells, while TIL-P and PBMC-NK cells showed 4.7- and 1.9-fold increases, reaching 39.0 × 10^3^ and 21.4 × 10^3^ NK cells, respectively (Figure 3A,B). Patients P1 and P2 demonstrated significantly higher NK cell expansion rates from TIL-C, with 61.5- and 121.6-fold increases compared to 10.3- and 5.4-fold in TIL-P, respectively. Although on day 12, the expansion rate of TIL-C was also superior to TIL-P, it decreased to 7.4-fold (Figure 3A). NK cell percentage in TIL remained stable at 5%, while significantly increased from 8.5% to 13.7% in PBMCs (Figure 3C). TIL-NK cell viability improved from 61% to 93% during expansion and remained stable at 85% in PBMCs (Figure 3D).

To further investigate the proliferation capacity of patient-derived NK cells under cytokine stimulation, we compared gene expression changes in expanded PBMC- and TIL-NK cells to NK cells isolated at the time of the surgery (PBMC-NK cells). Both expanded PBMC- and TIL-NK cells exhibited upregulation of key genes associated with cell proliferation. Specifically, expanded PBMC-NK cells showed upregulation of cytokine receptors (*IL2RA*), cell cycle regulation genes (*CDC6*, *CDK6*, *CDK1*, *CDKN1A*, *CCNB1*, *CCNA2*), and cell survival, proliferation and growth factor genes (*BIRC5*, *HBEGF*, and *CSF2RB*) (Figure 3E,F and Figure S4). Similarly, expanded TIL-C demonstrated upregulation of cytokine receptors (*IL2RA*), cell cycle regulation genes (*CDC6*, *CDK1*, and *CCNB1*), and cell survival, proliferation and growth factor genes (*BIRC5*, *HBEGF*, and *CSF2RB*) (Figure 3G, Figures S5 and S6). Expanded TIL-P also showed comparable upregulation patterns, differing only in the specific upregulated cell cycle genes (*CDK6*, *CDK1*, *CDKN1A*, *CCNB1*, *CCNA2*) (Figure 3H, Figures S7 and S8).

These results confirm that patient-derived NK cells can proliferate ex vivo under IL-2, IL-15, and brief IL-12 stimulation, with TIL-C demonstrating the highest expansion capacity.

### 3.5. IL-2, IL-15, and Brief IL-12 Stimulation Promotes Expansion of CD16- NK Cells

**Figure 4:**
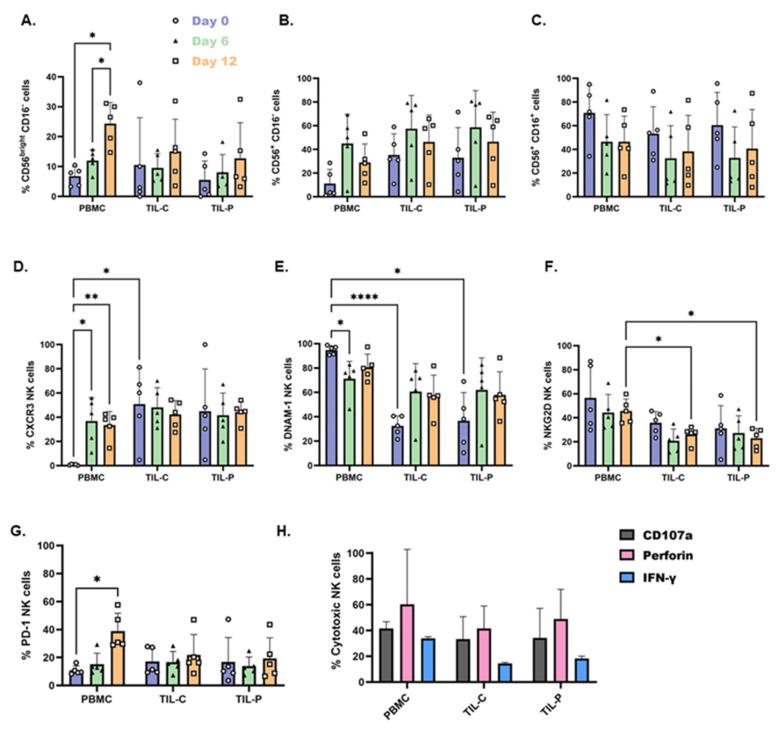
Phenotypical and functional characterization of patient-derived NK cells under IL-2, IL-15, and brief IL-12 stimulation. (**A**) Percentage of regulatory NK cell subset (CD56^bright^ CD16^−^) in total NK cells. (**B**) Percentage of intermediate NK cell subset (CD56^+^ CD16^−^) in total NK cells. (**C**) Percentage of cytotoxic NK cell subset (CD56^+^ CD16^+^) in total NK cells. Phenotypic analysis of (**D**) CXCR3, (**E**) DNAM-1, (**F**) NKG2D, and (**G**) PD-1 expression in NK cells. Each bar represents the mean and SD from 5 samples measured at day 0 (purple), day 6 (green), and day 12 (orange). Statistical differences were assessed using the ANOVA test (* *p* ≤ 0.05). (**H**) Functional analysis of NK cells from patients P3 and P5, measuring CD107a, perforin, and IFN-y levels in PBMC- and TIL-derived NK cells. Graphs represent the mean and SD (*n* = 2); CD107a is represented by grey bars, perforin by pink bars, and IFN-γ by blue bars. Statistical differences were assessed using a two-way ANOVA test, followed by Tukey’s multiple comparison test (* *p* ≤ 0.05, ** *p* ≤ 0.01, and **** *p* ≤ 0.0001).

The analyses of NK cell subsets showed that during culture, the percentage of regulatory NK cells (CD56^bright^ CD16^−^) increased across all NK cell sources, reaching 24.4% of total NK cells in PBMCs, and 15.1% and 12.7% in TIL-C and TIL-P, respectively (Figure 4A). Similarly, the percentage of intermediate NK cells (CD56^+^ CD16^−^) also increased in all cell sources compared to day 0 (Figure 4B), whereas the cytotoxic NK cell percentage (CD56^+^ CD16^+^) decreased during cell culture (Figure 4C). Patient P1 (who died of disease) and P5 (with advanced stage) exhibited the highest percentages of cytotoxic NK cells within TIL, with approximately 80% and 60%, respectively, while other patients showed less than 25%. Despite the high fractions of cytotoxic NK cells in these patients, there is a general enrichment of regulatory and intermediate (CD56^bright/+^ CD16^−^) NK cell subsets during cell culture. Nevertheless, stimulating patient-derived NK cells with these cytokines increased the CD56^bright^ NK cell proportion, potentially enhancing their tumor infiltration capacity.

### 3.6. Enhanced Tumor Infiltration, Activation, and Cytotoxicity Following Cytokine Stimulation

NK cell stimulation significantly modulated their phenotype and function. CXCR3 expression on PBMC-NK cells increased from nearly 0% to approximately 40%, while TIL-NK cells maintained stable expression at 50% (Figure 4D). This increase in CXCR3 expression on PBMC-NK cells, reaching similar levels to TIL-NK cells, suggests improved infiltration capacity, correlating with an increased presence of CD56^bright^ NK cells (Figure 4A). RNA sequencing revealed upregulation of key migration-related genes in both PBMCs and TIL-NK cells following cytokine stimulation compared to PBMC-NK cells at the time of the surgery. These included chemokine receptors (*CCR1*, *CXR1*, *XCL1*, *XCL2*, and *CCL3*) and extracellular matrix-modifying enzymes (FUT1, FUT7, and MMP14) (Figure 3E–H and Figures S4–S8). Expanded TIL-C additionally showed upregulation of cytokine receptor (ILR1), chemokine genes (*XCL1*, *XCL2*, and *CCL22*), and cell adhesion and migration gene (JAML) compared to expanded PBMC-NK cells (Figures S9 and S10). However, no significant differences were observed between TIL-NK cells from CTR and PTR.

Activation markers showed differential responses to cytokine stimulation. DNAM-1 expression slightly decreased in PBMC-NK cells but increased in TIL-NK cells, indicating improved activation (Figure 4E). Despite the decline of DNAM-1 in PBMCs, it remained higher than in TIL-NK cells, suggesting superior activation of PBMC-NK cells compared to TIL-NK (Figure 4E).

NKG2D expression decreased across all NK cell sources, with PBMC-NK cells (45.7%) maintaining higher levels than TIL-NK cells (~25%) (Figure 4F), further supporting superior activation in PBMC-NK cells. PD-1 expression increased in PBMC-NK cells (38.8%), particularly in patient P1 (~50%), but remained low in TIL-NK cells (Figure 4G).

RNA sequencing revealed upregulation of activation-associated genes in both PBMCs and TIL-NK cells following cytokine stimulation compared to PBMC-NK cells at the time of the surgery, such as cytokine and cytokine receptor genes (*IL2RA*, *IL23R*, *IL32*, *IL1R1*, and *IL33*) and costimulatory molecule genes (*CD70* and *CD86*) (Figure 3E–H and Figures S4–S8). Additionally, TIL-NK cells showed upregulation of *NCR3*, with TIL-C also demonstrating upregulation of activation gene *KLRF2* (Figure 3E,F and Figures S5–S8). No relevant genes were significantly downregulated in patient-derived NK cells after cell culture.

Cytotoxic markers were evaluated only in the two more advanced PDAC cases (P3 and P5), measuring CD107a (degranulation marker), perforin (cytotoxic molecule), and IFN-γ (inflammatory cytokine) levels. CD107a expression was similar across NK cell sources after cytokine stimulation (Figure 4H). However, PBMC-NK cells showed slightly higher levels of perforin and much higher IFN-γ production than TIL-NK cells (Figure 4H). RNA sequencing revealed upregulation of cytotoxicity-associated genes in patient-derived TIL, including *TNF*, *GZMA*, *GZMB*, and *IFNG* (Figure 3E–H and Figures S4–S8) compared to PBMC-NK cells at the time of the surgery.

Overall, stimulation with IL-2, IL-15, and brief IL-12 enhances the infiltration, activation, and cytotoxic potential of patient-derived NK cells, particularly TIL-NK cells from the CTR. However, PBMC-NK cells exhibit superior cytotoxic capacities compared to TIL-NK cells.

## 4. Discussion

TILs provide crucial insights into the prognosis and survival of cancer patients [35]. While most studies in TIL therapy focus on T-cells, NK cells in the TME have been associated with improved overall survival [36]. Nevertheless, exploiting tumor-infiltrating NK cells faces challenges due to limited expansion capabilities, low cell availability, and a lack of comprehensive understanding of NK cells and their subsets within the TME [37]. Here, we evaluated NK cell phenotype, expansion rates, tumor infiltration, and cytotoxic capacities, while comparing patient-derived NK cells from the central and peripheral tumor regions and those circulating in the PB of five patients with PDAC.

Our findings revealed that PTR exhibited significantly superior NK cell infiltration compared to CTR. This difference can be a consequence of the PDAC immunosuppressive environment with dense fibrotic stroma that challenges the infiltration of immune cells [38]. Interestingly, the two more advanced PDAC cases displayed a higher percentage of NK cells infiltrating the tumor center, suggesting superior infiltration capacities. As expected, since PDAC is mostly described as a “cold” tumor, and in concordance with previous studies, patient-derived PBMCs displayed a higher fraction of NK cells compared to TIL [39,40].

The cytotoxic NK cell subset was predominant in both PBMC- and TIL-derived NK cells; however, CTR displayed enrichment of CD16^−^ NK cells, especially in more advanced and aggressive tumor. Previous findings have reported increased CD16^−^ NK cells in tumors, describing that metalloprotease-17 (ADAM17) can shed CD16 in activated NK cells, leading to a soluble form that competes for antibody binding, reducing NK cell cytotoxicity [39,41,42]. In our study, TIL-NK cells, isolated at the time of the surgery, exhibited lower DNAM-1 and NKG2D but higher CXCR3 expression than PBMC-NK cells, suggesting potential inferior cell activation but higher tumor infiltration capacities. Additionally, TIL-NK cells displayed lower NKp30, CD57, and CXCR2 expression than PBMCs in other studies, supporting NK cell dysfunction [39,40].

Expanding NK cells ex vivo remains challenging, especially from solid malignancies [43]. Here, we described a successful protocol to isolate and culture NK cells from PDAC specimens without enzymatic digestion, ensuring cell viability, using feeder cells and cytokines for NK cell expansion. From over 26 cytokine combinations tested in healthy donors and three tested in patient-derived NK cells the IL-2, IL-15, and brief IL-12 exposure promoted the highest cytotoxic NK cell expansion rates. IL-2 and IL-15 are both γ-chain cytokines, stimulating mTOR and STAT5 pathways, having an important role in cell survival and proliferation, while IL-12 stimulates the STAT4 pathway, promoting cytokine production, enhancing IFN-γ production and cytotoxicity [44,45,46,47,48]. In our study, TIL-NK cells displayed higher expansion rates than PBMC-derived NK cells, particularly TIL-C, indicating that even in a highly immunosuppressive TME, NK cells maintain expansion and proliferation capacities. Expanded TIL-NK cells showed improved cell activation with increased DNAM-1 expression compared to NK cells at the time of the surgery. Also, the activation and infiltration capacities of PBMC-NK cells improved during culture, showing increased levels of DNAM-1, NKG2D, and CXCR3. Indeed, CXCR3 expression in PBMC-NK cells reached similar levels to those in TIL-NK cells on day 12. Increased levels of CXCR3 and CXCR4 have been previously reported in PBMC-NK cells facilitating their migration into the tumor [49]. Here, both PBMC and TIL-NK cells displayed upregulation of key genes associated with cell proliferation, including *IL2RA* and *CSF2RB* mediating cytokine signaling pathways through IL-2, IL-3, IL-5 and GM-CSF; *CDC6*, *CDK6*, *CDK1*, *CDKN1A*, *CCNB1*, and *CCNA2* promoting cell cycle progression; *BIRC5* inhibiting apoptosis and promoting survival; and *HBEFG* promoting cell growth and proliferation through EGF-like growth factors.

In lung cancer, TIL-NK cells are predominantly CD56^bright^ perforin^low^, which exhibit lower cytotoxic potential, but similar cytokine production compared to PBMC-NK cells [50]. Consistently, we observed higher infiltration of CD56^bright^ NK cells in the CTR compared to the PTR, indicating their superior tumor infiltration capacity. PBM- and TIL-NK cells, particularly TIL-C, exhibited upregulation of genes involved in cell migration. These include chemokine receptors such as *CCR1* which plays a role in recruiting and activating macrophages and neutrophils to the TME [51]; *CXR1*, which promotes leukocyte chemotaxis to sites of inflammation [52]; *XCL1* and *XCL2*, which help chemotaxis by interacting with dendritic cells (DCs) [53]; *FUT1* and *FUT7*, which are linked to immune cell infiltration in the tumor [54,55]; and *MMP14*, which plays a role in the breakdown and remodeling of the extracellular matrix [56]. Additionally, when comparing expanded PBMC and TIL-NK cells, the latter showed superior expression of *IL1R*, which mediates immune responses through NF-kB and MAPK pathways [57]; *XCL1*, *XCL2*, and *CCL22*, which is involved in recruiting DCs to tumors [53]; and *JAML* which plays a role in immune cell adhesion and migration [58].

TIL-NK cells in some cancers displayed low cytotoxicity [50,59]. In our study, we consistently observed in the five patients that NK cell activation and cytotoxicity were improved after cytokine stimulation. Furthermore, PBMC and TIL-NK cells revealed upregulation of genes associated with NK cell activation and cytotoxicity, including *IL2RA* and *IL23R*, binding to IL-2 and IL-23 and stimulating IFN-γ secretion [60]; *IL1RL1* and *IL33* contributed to NK cell activation via cooperation with IL-12 [61]; *CD70* was associated with increased IFN-γ production through AKT signaling pathways [62]; *IL3,2* was linked to enhanced NK cell cytotoxicity [63]; *CD86* was associated with the improved killing capacity of NK cells [64]; and *GZMA*, *GZMB*, and *TNF*, which are cytotoxic molecules secreted by NK cells [65]. In addition, TIL-C also revealed upregulation of *KLRF2* and *NCR3* coding for NK cell activation markers, NKp65 and NKp30, respectively [66,67]; *IFNG* was involved in NK cell targeting and killing cancer cells [68].

Sequencing data also revealed upregulation of genes associated with cell proliferation, tumor infiltration capacity, activation, and cytotoxicity in PBMC-NK cells. These results emphasize the potential of PBMCs as a source of NK cells for adoptive cell therapy due to their higher availability and strong activation and cytotoxicity, especially after ex vivo stimulation. TIL-NK cells displayed significantly higher proliferation capacity than PBMC-NK cells, which is a great advantage for adoptive cell therapies.

Some limitations of this pilot work cannot be ignored: the restricted inclusion criteria allowed the collection and analysis of only five PDAC patient samples in a timely manner. Furthermore, the limited size of the collected fresh specimens (not to impair clinical diagnosis) resulted in low starting cell numbers, preventing the achievement of relevant NK cell numbers, which did not permit the performance of in vitro and in vivo functional studies, which would have added critical information to this report. Additionally, this study focused on the comparison between PBMCs and TIL-derived NK cells from each patient, and there is no data on “normal adjacent” pancreas tissue, which could have served as a tissue “control”. Finally, although TIL-NK cells reached a higher fold change after 6 days, they dropped on day 12, in contrast to PBMC-derived NK cells.

## 5. Conclusions

This pilot study, although preliminary, provides crucial knowledge on PDAC-infiltrating NK cells and demonstrated that cytotoxic NK cells were the most present subtype in PDAC patients, both in PB- and tissue-derived TILs. Notably, PBMC-derived NK cells showed higher activation but lower tumor infiltrating capacity than TIL-derived NK cells. Moreover, NK cells in the central tumor regions showed higher infiltrating capacity than the ones in the periphery. After cytokine stimulation, the proliferation capacity, cell activation and cytotoxicity of TIL-NK cells significantly increase, which make them very suitable for adoptive cell therapy. Although further studies are needed with more patients, this work offers a first understanding of NK cells and their subsets in PDAC TME and PB and holds significant implications for advancing tailored cellular immunotherapies.

## Figures and Tables

**Table 1:** Primary antibodies used for flow cytometry and immunofluorescence analyses.

Marker	Company	Catalog Number
CD45-Alexa700	Biolegend	304024
CD19-APC/Cy7	Biolegend	363010
CD3-PE/Cy7	BD	563423
CD4-PB	Beckman	B49197
CD8-KrO	Beckman	B00067
CD56-PE	BD	345812
CD16-BV785	Biolegend	360734
PD-1-PerCP/Cy5	Biolegend	329914
NKG2D-PE/CF594	BD	562498
CXCR3-FITC	Biolegend	353703
DNAM-1-Alexa647	Biolegend	338327
viability dye	Invitrogen	L34959
CD107a-APC	BD	641581
IFN-γ-FITC	BD	561057
perforin-PerCP/Cy5.5	BD	563762
CD3 [CD3-12]	Abcam	ab11089
CD16 [2H7]	GeneTex	GTX7539
NKG2D [EPR24072-342]	Abcam	302907
cytokeratin18 (CK18)	Abcam	ab219271
AF488 NCAM1 (CD56) [EP2567Y]	Abcam	ab237455

**Table 2:** Clinico-pathological information of patients.

Patient	P1	P2	P3	P4	P5
**Sex**	F	F	F	M	M
**Age at surgery**	72	90	78	67	71
**Diagnosis at surgery**	IPMN associated PDAC	IPMN associated PDAC	IPMN associated PDAC	IPMN associated PDAC	PDAC
**pT**	2	1c	3	2	2
**pN**	1	1	2	0	2
**pM**	0	0	0	0	0
**p stage**	IIB	IIB	III	IB	III
**R status**	0	0	0	0	0
**LVI**	yes	yes	yes	no	no
**PN inv**	yes	yes	yes	yes	yes
**V inv**	yes	no	yes	no	yes
**MMR**	MSS	MSS	MSS	MSS	MSS
**Her2**	negative	negative	negative	negative	negative
**BRAF**	negative	negative	negative	negative	negative
**KRAS**	negative	negative	negative	negative	negative
**Metastasis**	1	0	1	0	0
**Site of M1**	Liver	0	Lung/Peritoneu	0	0
**Follow-up (months)**	15	18	16	16	17
**Status**	DOD	AWOD	AWD	AWOD	AWOD

Patient (P); female (F); male (M); pancreas ductal adenocarcinoma (PDAC); intraductal papillary mucinous neoplasm (IPMN); tumor (T); node (N); metastasis (M); pathological stage (p stage); resection status (R status); lymphovascular invasion (LVI); perineural invasion (PN inv); venous invasion (V inv); mismatch repair (MMR); died of disease (DOD); alive with disease (AWD); alive without disease (AWOD).

## Data Availability

Sequencing data that support the findings of this study have been deposited in the EBI European Nucleotide Archive with the primary accession code PRJEB68083.

## Supplementary Materials

The following supporting information can be downloaded at: https://www.mdpi.com/article/10.3390/cells15090797/s1, Figure S1: Analysis of NK cells in PBMCs and tumor specimens from each patient at surgery; Figure S2: Analysis of NK cell subsets in PBMCs and tumor specimens from each patient at surgery; Figure S3: Phenotypic characterization of NK cells in PBMCs and tumor specimens from each patient at surgery; Figure S4: Functional enrichment analysis of upregulated genes in expanded PBMC-NK compared to PBMC-NK at surgery; Figure S5: Functional enrichment analysis of upregulated genes in expanded TIL-C compared to PBMC-NK at surgery; Figure S6: Transcriptional profiling of expanded TIL-C compared to PBMC-NK at surgery; Figure S7: Functional enrichment analysis of upregulated genes in expanded TIL-P compared to PBMC-NK at surgery; Figure S8: Transcriptional profiling of expanded TIL-P cells compared to PBMC-NK at surgery; Figure S9: Functional enrichment analysis of upregulated genes in expanded TIL-C compared to expanded PBMC-NK; Figure S10: Transcriptional profiling of expanded TIL-C compared to expanded PBMC-NK.
