# Supplementary material for: Tumor-Infiltrating Natural Killer Cell Characterization in Pancreatic Ductal Adenocarcinoma
Source: Cells. 2026 Apr 28;15(9):797. doi: 10.3390/cells15090797 (PMC13162876; doi:10.3390/cells15090797)

SUPPLEMENTARY MATERIAL LIST

Figure S1. Analysis of NK cells in PBMCs and tumor specimens from each patient at surgery. **(A)** Quantification of NK cell numbers in 9mL of peripheral blood and fresh tumor specimens. **(B)** Percentage of NK cells in total lymphocytes. **(C)** Quantification of NK cell number per mm^2 in fixed tumor specimens. The first graph represents the mean and SD (n=5), while the P1 to P5 graphs represent individual patient values; PBMCs are represented by blue bars, TIL-C by darker green, and TIL-P by lighter green bars. Statistical differences were assessed using ANOVA or t-test *$p \leq 0.05$, **$p \leq 0.01$, ***$p \leq 0.001$.

Figure S2. Analysis of NK cell subsets in PBMCs and tumor specimens from each patient at surgery. **(A)** Percentage and **(B)** cell number per mm^2 of regulatory ($CD56^{bright}$ $CD16^-$) NK cells. **(C)** Percentage of intermediate ($CD56^+$ $CD16^-$) NK cells. **(D)** Percentage and **(E)** cell number per mm^2 of cytotoxic ($CD56^+$ $CD16^+$) NK cells. The first graph represents the mean and SD (n=5), while the P1 to P5 graphs represent individual patient values; PBMCs are represented by blue bars, TIL-C by darker green, and TIL-P by lighter green bars. Statistical differences were assessed using ANOVA or t-test *$p \leq 0.05$.

Figure S3. Phenotypic characterization of NK cells in PBMCs and tumor specimens from each patient at surgery. Analyses of **(A)** CXCR3, **(B)** DNAM-1, and **(C)** NKG2D expression on NK cells. **(D)** Quantification of $NKG2D^+$ NK cell numbers per mm^2 in tumor specimens. **(E)** PD-1 expression on NK cells. The first graph represents the mean and SD (n=5), while the P1 to P5 graphs represent individual patient values; PBMCs are represented by blue bars, TIL-C by darker green, and TIL-P by lighter green bars. Statistical differences were assessed using ANOVA or t-test *$p \leq 0.05$, **$p \leq 0.01$, and ***$p \leq 0.001$.

Figure S4. Functional enrichment analysis of upregulated genes in expanded PBMC-NK compared to PBMC-NK at surgery. Enriched biological pathways in PBMC-NK cells following 12 days of stimulation with IL-2, IL-15, and brief IL-12. The bar graph illustrates the number of upregulated genes associated with each enriched pathway. The specific number of genes is indicated next to each bar.

Figure S5. Functional enrichment analysis of upregulated genes in expanded TIL-C compared to PBMC-NK at surgery. Enriched biological pathways in TIL-C following 12 days of stimulation with IL-2, IL-15, and brief IL-12. The bar graph illustrates the number of upregulated genes associated with each enriched pathway. The specific number of genes is indicated next to each bar.

Figure S6. Transcriptional profiling of expanded TIL-C compared to PBMC-NK at surgery. Heatmap illustrating differentially expressed genes in NK cells isolated from the central tumor region (TIL-C) following 12 days of stimulation with IL-2, IL-15, and brief IL-12, compared to PBMC-NK cells isolated at the time of the surgery.

Figure S7. Functional enrichment analysis of upregulated genes in expanded TIL-P compared to PBMC-NK at surgery. Enriched biological pathways in TIL-P following 12 days of stimulation with IL-2, IL-15, and brief IL-12. The bar graph illustrates the number of upregulated genes associated with each enriched pathway. The specific number of genes is indicated next to each bar.

Figure S8. Transcriptional profiling of expanded TIL-P cells compared to PBMC-NK at surgery. Heatmap illustrating differentially expressed genes in NK cells isolated from the

peripheral tumor region (TIL-P) following 12 days of stimulation with IL-2, IL-15, and brief IL-12, compared to PBMC-NK cells isolated at the time of the surgery.

Figure S9. Functional enrichment analysis of upregulated genes in expanded TIL-C compared to expanded PBMC-NK. Enriched biological pathways in TIL-C following 12 days of stimulation with IL-2, IL-15, and brief IL-12 compared to PBMC-NK cells under the same stimulation conditions. The bar graph illustrates the number of upregulated genes associated with each enriched pathway. The specific number of genes is indicated next to each bar.

Figure S10. Transcriptional profiling of expanded TIL-C compared to expanded PBMC-NK. Heatmap illustrating differentially expressed genes in NK cells isolated from the central tumor region (TIL-C) following 12 days of stimulation with IL-2, IL-15, and brief IL-12, compared to PBMC-NK cells stimulated at the same conditions.

Figure S1. Analysis of NK cells in PBMCs and tumor specimens from each patient at surgery.

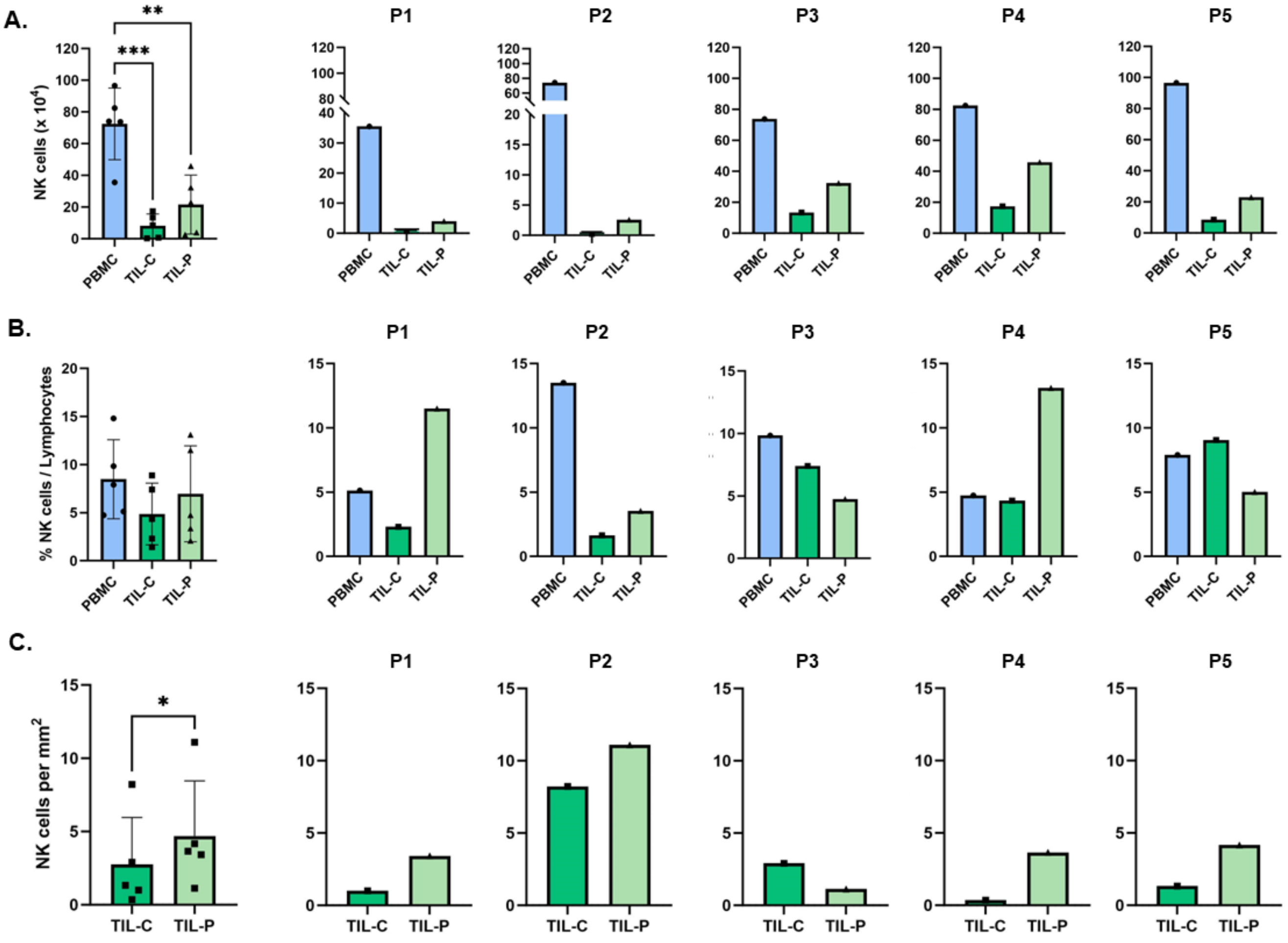

Figure S2. Analysis of NK cell subsets in PBMCs and tumor specimens from each patient at surgery.

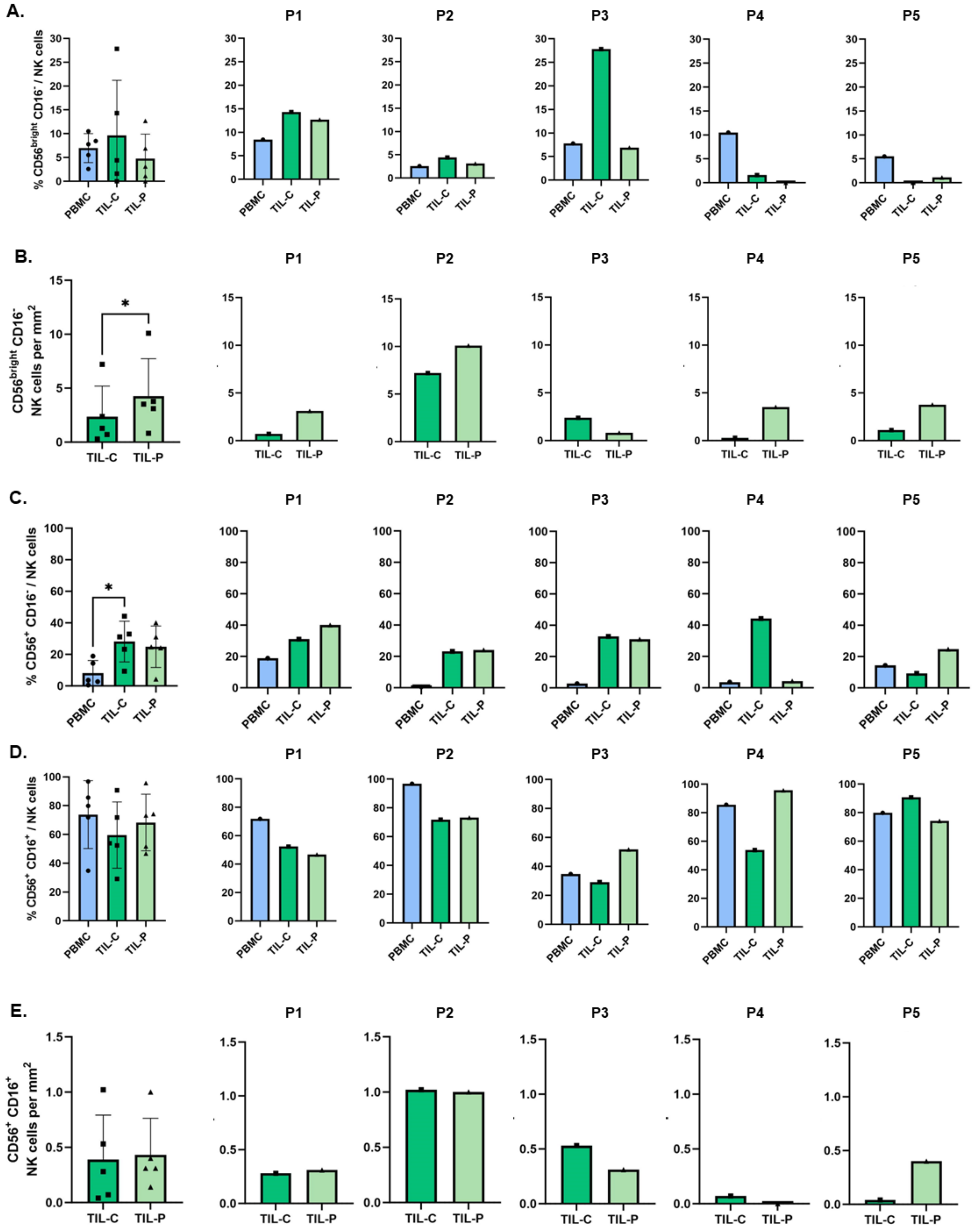

Figure S3. Phenotypic characterization of NK cells in PBMCs and tumor specimens from each patient at surgery.

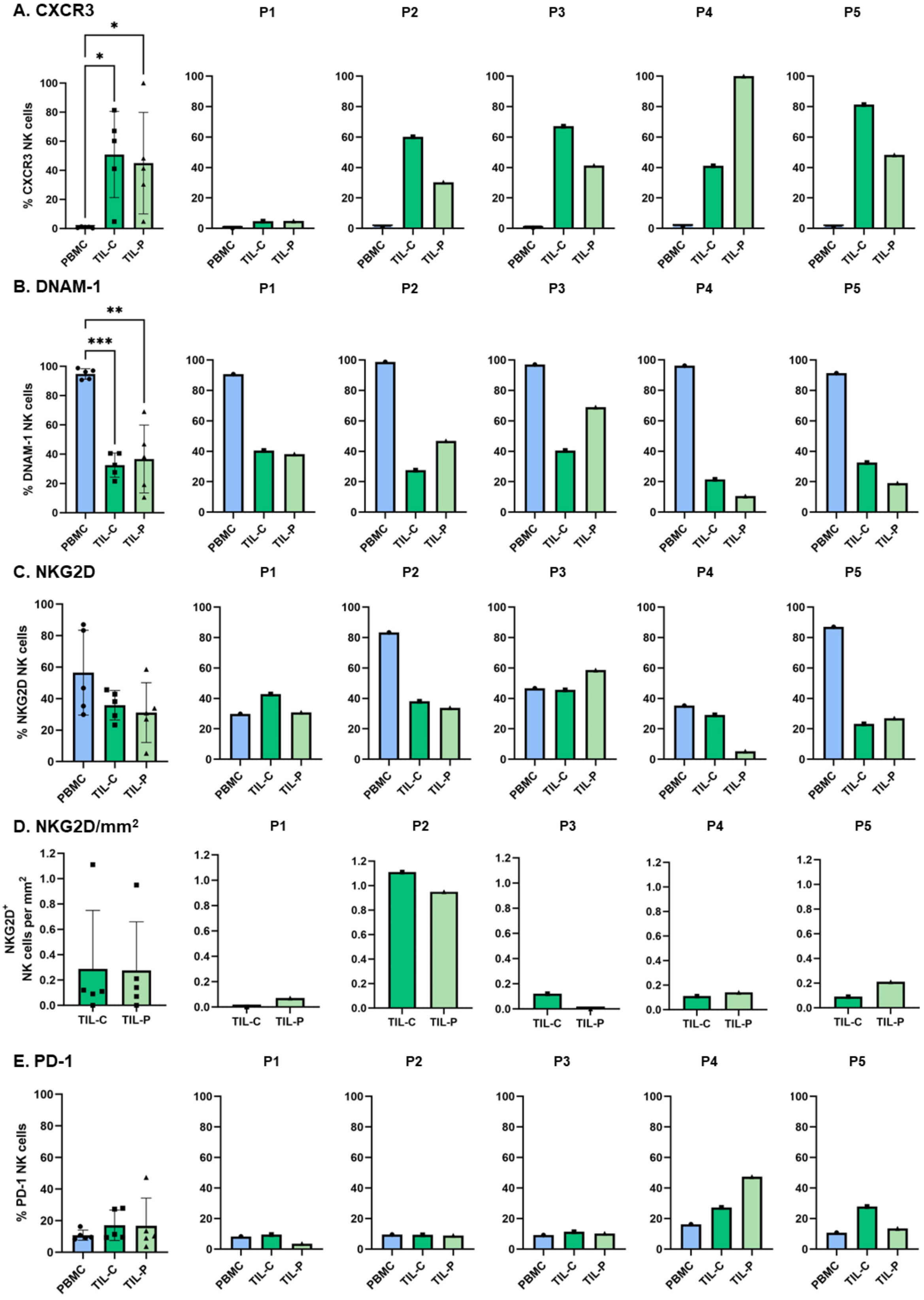

Figure S4. Functional enrichment analysis of upregulated genes in expanded PBMC-NK compared to PBMC-NK at surgery.

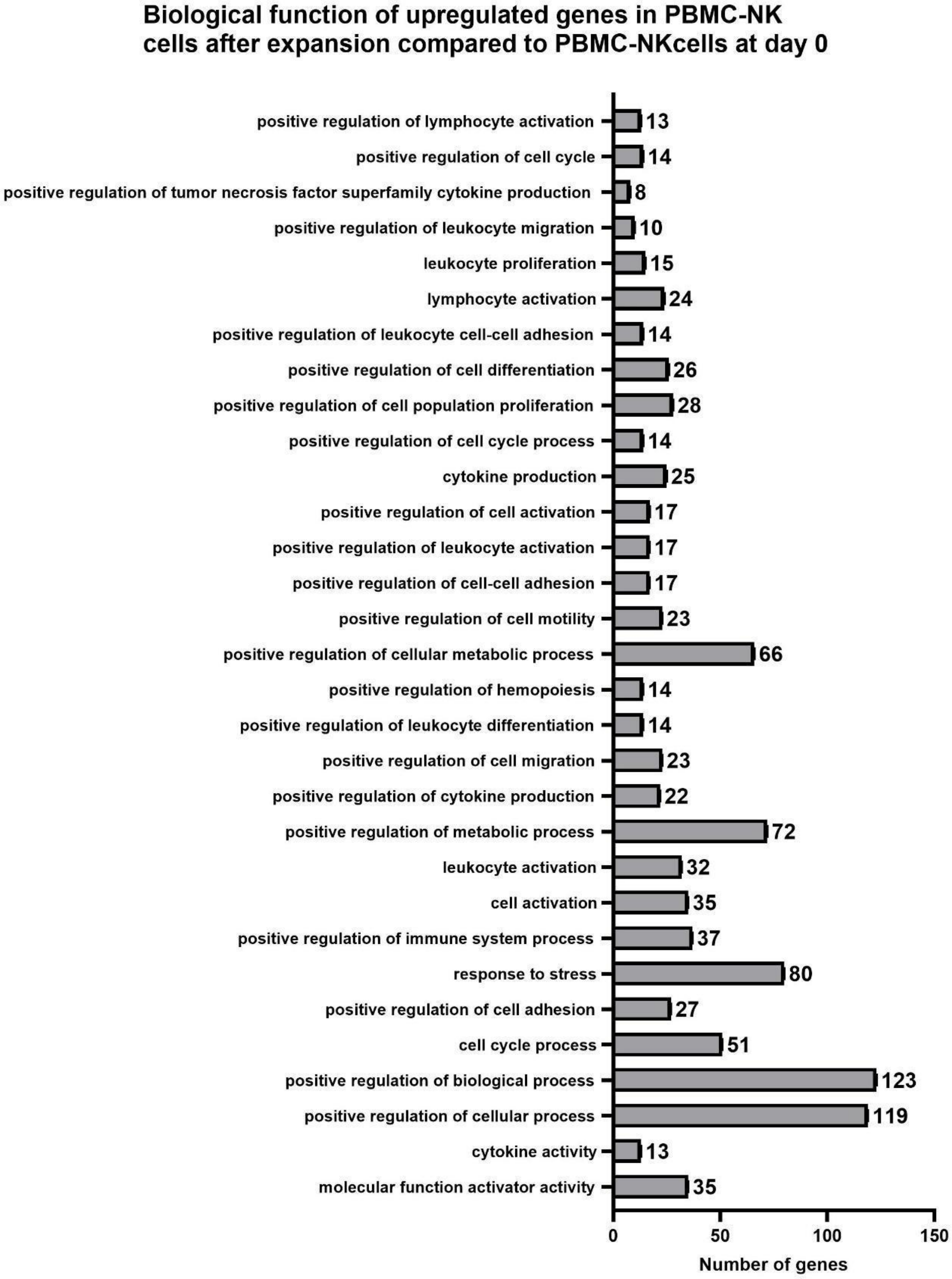

Figure S5. Functional enrichment analysis of upregulated genes in expanded TIL-C compared to PBMC-NK at surgery.

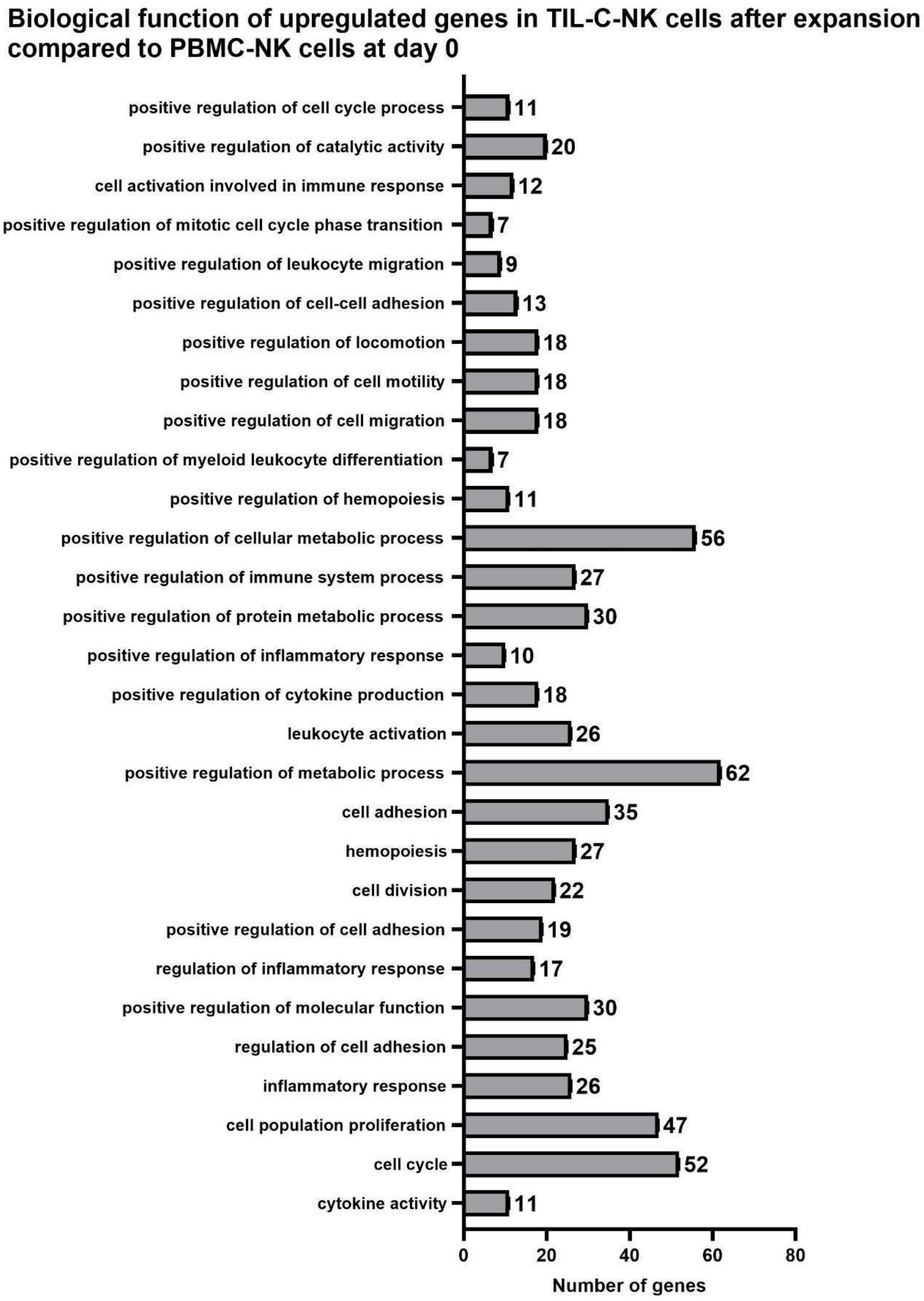

Figure S6. Transcriptional profiling of expanded TIL-C compared to PBMC-NK at surgery.

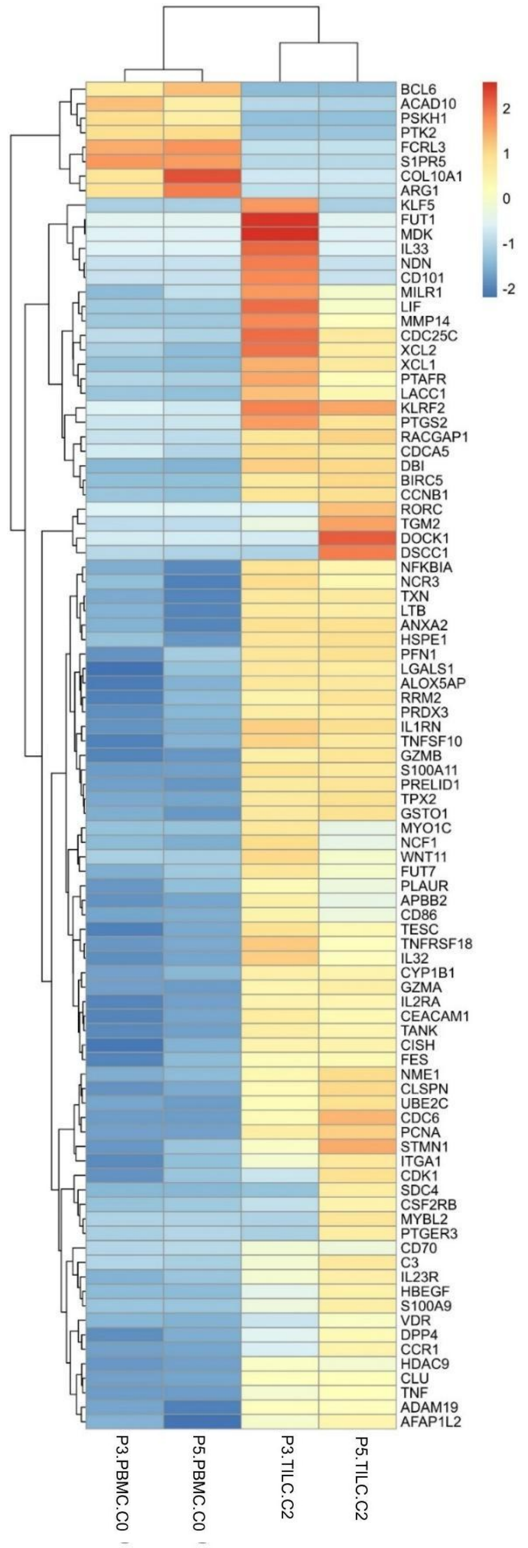

Figure S7. Functional enrichment analysis of upregulated genes in expanded TIL-P compared to PBMC-NK at surgery.

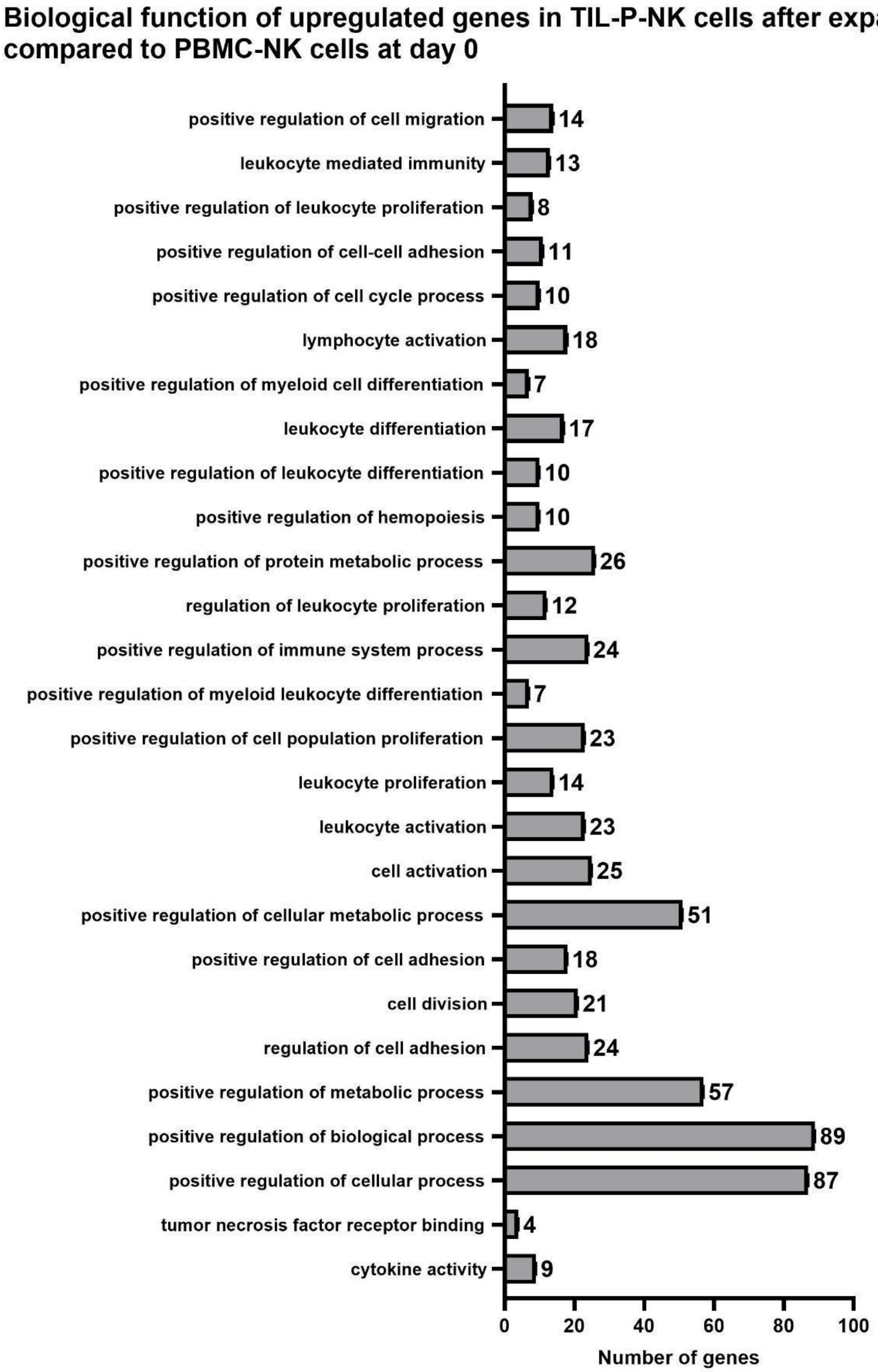

Figure S8. Transcriptional profiling of expanded TIL-P cells compared to PBMC-NK at surgery.

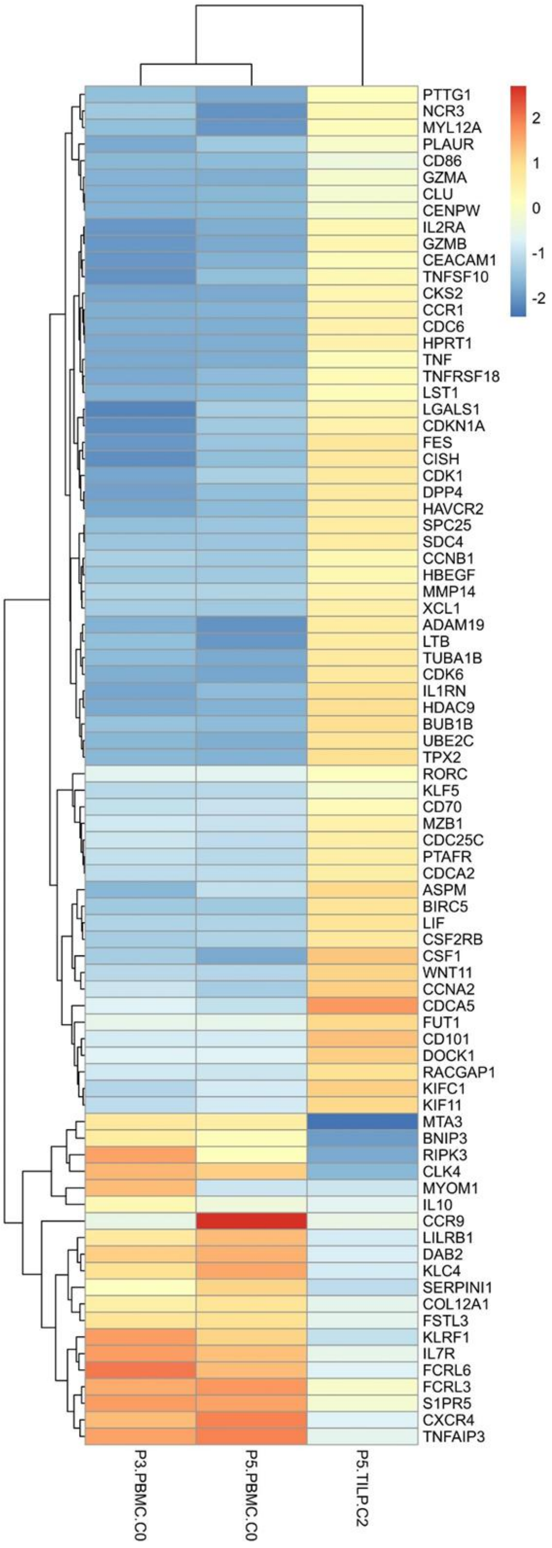

Figure S9. Functional enrichment analysis of upregulated genes in expanded TIL-C compared to expanded PBMC-NK.

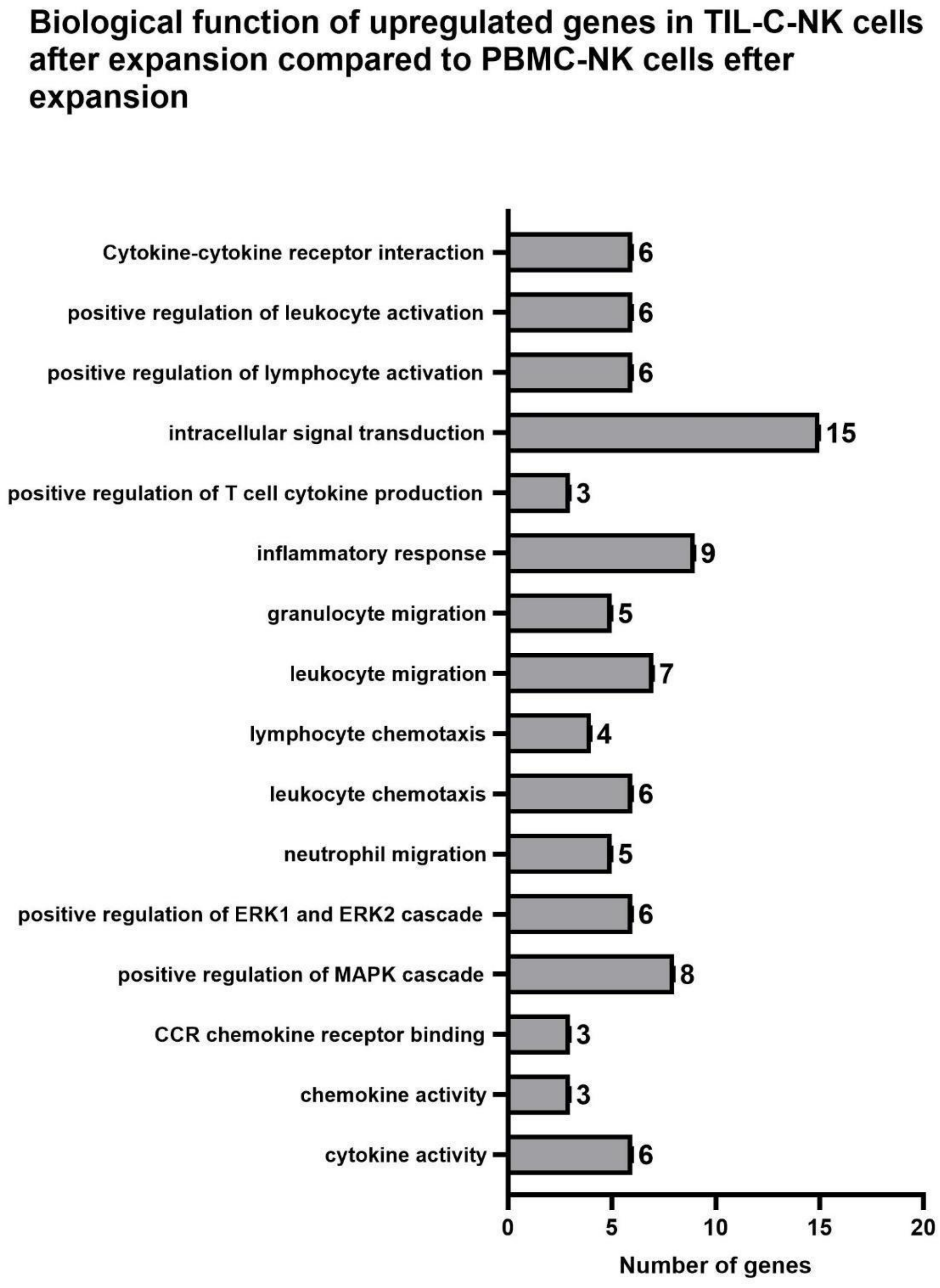

Figure S10. Transcriptional profiling of expanded TIL-C compared to expanded PBMC-NK.

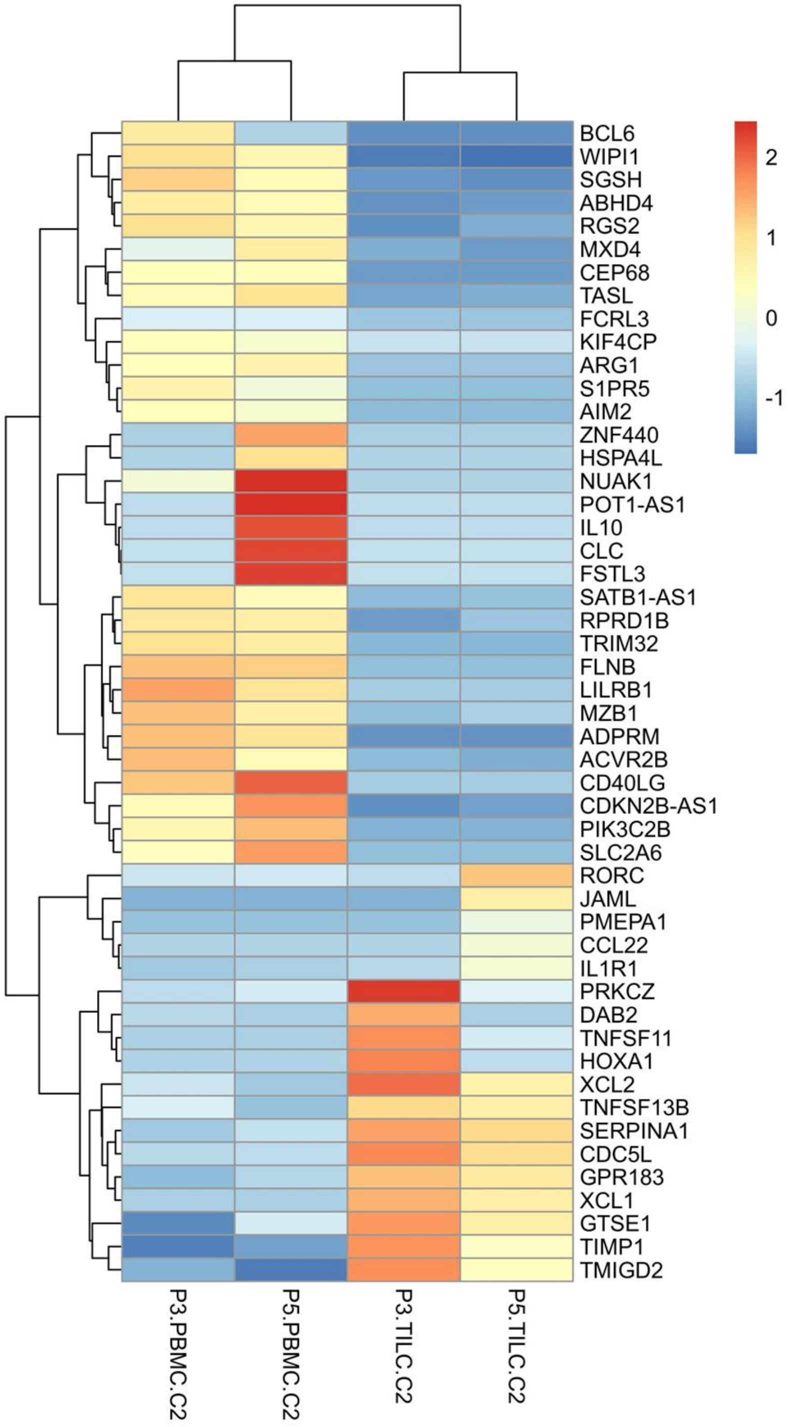

Supplement: Supplementary file 1 [file cells-15-00797-s001.zip › cells-4197776-supplementary.pdf]
